# Advanced Strategies Against SARS-CoV-2 Variants and Future Emerging Virus Outbreaks

**DOI:** 10.3390/v18010001

**Published:** 2025-12-19

**Authors:** Jacques Fantini

**Affiliations:** Department of Biology, Faculty of Medicine, Aix-Marseille University, INSERM UA16, 13015 Marseille, France; jacques.fantini@univ-amu.fr

## 1. Introduction

The COVID-19 pandemic, unleashed by SARS-CoV-2 in 2019 and amplified by variants such as Omicron, exposed global vulnerabilities, with over 700 million cases and 7 million deaths, fueling persistent fears of future pandemics from zoonotic RNA viruses amid accelerating urbanization and climate-driven spillovers [1]. Mpox epidemics and Hand, Foot, and Mouth Disease (HFMD), which have seen surges across Asia and the Americas, also necessitate rapid worldwide reactivity to contain spread and mitigate impacts from potential pandemics [2,3]. The articles in this Special Issue collectively illuminate advanced strategies to combat emerging viral threats—including SARS-CoV-2 variants, Mpox, and HFMD viruses—and to enhance preparedness for future outbreaks through advances in genomic surveillance and innovative therapeutics. These contributions underscore the imperative for integrated, interdisciplinary efforts in virology, emphasizing the rapid adaptation of vaccines and diagnostics amid evolving threats. Ultimately, sustained global collaboration will fortify resilient health systems, transforming insights from these works into proactive defenses against emerging pandemics.

## 2. SARS-CoV-2

Identifying universal antivirals against SARS-CoV-2 variants has proven essential, as relentless Spike protein mutations have evaded targeted therapies, demanding broad-spectrum solutions resilient to viral evolution. The N-terminal domain (NTD) of the Spike protein anchors the virus by binding gangliosides within cholesterol-rich lipid rafts on host membranes, facilitating subsequent ACE2 engagement and entry—a mechanism conserved across variants [4]. Lefebvre et al. [5] demonstrate ivermectin’s mutation-tolerant binding to the NTD. They developed dedicated flexible docking protocols, employing Polak–Ribière minimization in HyperChem to iteratively optimize ligand and protein conformations. This yields superior binding energies over rigid methods, capturing lipid raft dynamics. These in silico approaches were validated by wet-lab microtensiometry experiments revealing ivermectin-mediated spike displacement from ganglioside membranes. These findings reveal a novel antiviral action through the competitive inhibition of initial NTD–ganglioside interactions in raft microdomains.

Studies on hybrid immunity (infection + vaccination) are vital in informing booster strategies amid evolving variants and waning protection. The study by McConney et al. [6] tracks antibody responses in 46 individuals infected with SARS-CoV-2 in Spring 2020, measuring RBD- and N-specific IgG/IgA via a dedicated ELISA method (BU ELISA) [7] and neutralization against SARS-CoV-2 variants (Delta and Omicron) up to two years post symptom onset. They show that pre-vaccination, anti-N antibodies waned faster than anti-RBD, but post-vaccination (an average 8 months after infection), RBD IgG/IgA surged and stayed elevated for over one year, with no significant decline. These data suggest that infection followed by vaccination yields robust, durable responses lasting one year, supporting annual boosters to maintain protection.

Li et al. [8] identified a three-gene T-cell signature—CXCL10, IL-32, and PRF1—that differentiates SARS-CoV-2 infection from common respiratory viruses such as influenza and RSV with high accuracy. These researchers analyzed bulk PBMC RNA-seq from 113 patients, revealing elevated CXCL10 (chemokine), IL-32 (pro-inflammatory cytokine), and PRF1 (perforin for cytotoxicity) transcripts specifically in COVID-19 T cells versus other viral pneumonias. Single-cell validation confirmed CD8+ T-cell enrichment, distinguishing severe COVID from flu/RSV cohorts. The signature enables rapid blood-based diagnosis via RT-qPCR, aiding in triage and antiviral allocation amid variant surges and outperforming clinical scores alone. Such studies are crucial in developing precise diagnostic tools that differentiate SARS-CoV-2 from other respiratory viruses, enabling targeted clinical management and improved patient outcomes even in asymptomatic cases.

The emergence of SARS-CoV-2 has led to significant interest in its potential transmission between animals and humans, especially pets. Abay et al. [9] examine SARS-CoV-2 infections across wild and companion animals, emphasizing reverse zoonosis from humans and advocating for a One Health approach to mitigating public health risks. Cats, dogs, ferrets, and zoo species such as tigers show susceptibility via close human contact, with predominantly mild symptoms but the potential for transmission among animals. Primary spread occurs from infected owners to pets (e.g., cats develop respiratory signs), with limited pet-to-human risk but documented mink farm spillovers. Factors such as local COVID-19 rates influence animal cases; surveillance gaps hinder full assessment. The authors stress integrated human–animal-environment monitoring, pet precautions, and research into reservoirs to prevent future pandemics. Enhanced veterinary diagnostics and awareness campaigns are recommended.

The early identification of individuals at increased risk for a severe COVID-19 clinical course is crucial in enabling the timely initiation of appropriate therapy. In their comprehensive review, Jonjić et al. [10] synthesize evidence linking gut microbiota dysbiosis to COVID-19 severity, highlighting reduced microbial and the enrichment of opportunistic pathogens as predictors of poor outcomes. Their analysis underscores a close relationship between gut microbiota composition and disease progression, revealing distinct microbial signatures in patients with a critical clinical course. These findings suggest that gut microbiota composition markers could serve as valuable tools for early risk assessment and that the targeted modulation of the gut microbiota may represent a promising strategy to prevent severe manifestations and fatal outcomes in COVID-19.

Wastewater RNA levels provide an early, unbiased indicator of SARS-CoV-2 circulation in communities, preceding clinical case surges by 6–8 days to enable proactive public health responses. Armenta-Castro et al. [11] analyzed Mexican COVID-19 trends using wastewater surveillance data processed via simple machine learning algorithms for rapid public health decisions. They conclude that while this approach can be used for simple risk assessment for the deployment of adequate prevention and containment strategies within high-affluence facilities, such as universities or workspaces, especially in the early stages of a possible epidemiological outbreak, further work is still needed toward the integration of more robust datasets into more complex models capable of long-term forecasting that could be used for future pathogens similar to COVID-19.

Bala et al. [12] measured cathepsin protease activity and protein levels in urine samples from COVID-19 patients and controls, identifying C-reactive protein, cathepsins S and L, and klotho enrichment in urinary extracellular vesicles (uEVs) as potential biomarkers. Proteomic pathway analyses revealed that most identified proteins were involved in stress responses, protein metabolism, and transport. These proteins were mainly associated with cellular membranes and functions related to the cytoskeleton, enzyme regulation, and signal transduction. Collectively, these urinary markers provide non-invasive insights into SARS-CoV-2-induced renal effects and possible long-term sequelae, warranting further validation for clinical monitoring.

## 3. Mpox

Mpox virus (formerly monkeypox virus) has emerged as a significant public health concern due to its global outbreaks since 2022. Yadav et al. [13] provide a comprehensive review of its epidemiology from February 2022 to April 2025, documenting 97,281 confirmed cases across 118 countries by June 2024—prompting a WHO public health emergency declaration in August 2024. Covering replication, genomics, pathology, close-contact transmission (including sexual), PCR diagnosis, and therapeutics such as tecovirimat, cidofovir, and ribavirin, alongside supportive care, the analysis details symptoms (fever, headache, myalgias, lymphadenopathy, and rash progressing to pustules, often anogenital in clade IIb), complications (superinfections, pneumonia, encephalitis, proctitis, and ocular issues), and heightened risks in HIV-coinfected patients. Modified vaccinia Ankara vaccination and Orthopoxvirus antibodies offer cross-protection, underscoring integrated prevention, while drug repositioning merits consideration, as previously suggested by Wu et al. [14].

Bragazzi et al. [15] offer a comprehensive global review of Mpox infections in cisgender women, transgender women, and non-binary individuals assigned female at birth. Documented across regions including Argentina, Brazil, Europe, Nigeria, the US, and Vietnam, these cases reveal distinct epidemiological and clinical patterns compared to men, including delayed diagnoses, psychological impacts, and heightened risks for pregnant women or those with comorbidities. By critiquing biases in predominantly male-focused reporting—paralleling SARS-CoV-2 disparities—the study calls for gender-inclusive surveillance, genomic sublineage monitoring, and tailored public health strategies to address knowledge gaps and enhance outbreak control.

The 2022 global Mpox outbreak swiftly introduced unforeseen diversity into the monkeypox virus (MPXV) population, necessitating the revalidation of primers against thousands of genomes to ensure diagnostic robustness. Song et al. [16] evaluated 18 PCR primer sets across 5210 MPXV genomes spanning all lineages, underscoring the need for lineage-specific monitoring, as mutations impair sensitivity in low-viral-load samples such as saliva or urine. Top performers, such as F3L_M and E9L primers targeting highly conserved segments, offer robust qPCR options for clinical laboratories, while analysis revealed 173 conserved regions (>150 bp) for future designs. This in silico framework ensures that detection schemes are robust and guides primer evaluation for other emerging infectious diseases.

## 4. Hand, Foot, and Mouth Disease (HFMD)

HFMD, primarily caused by enterovirus A71 and coxsackievirus A16, exemplifies an emerging viral outbreak through its recurrent large-scale epidemics in the Asia-Pacific region. Ravel [17] et al. analyzed the first HFMD outbreak in North Vietnam (Hải Phòng, summer 2011–autumn 2012), developing a mathematical model that accurately fitted the total cases and weekly trends, pinpointing overlapping epidemic waves and patient-wave probabilities. This framework enables real-time forecasting to guide interventions and extends to other diseases, preempting escalations akin to recent Orthopoxvirus events.

## 5. Conclusions

Virus outbreaks such as COVID-19 (SARS-CoV-2 variants), recent Mpox epidemics, and HFMD surges demonstrate the need for rapid worldwide reactivity. Combining in silico approaches with wet-lab experiments enables rational therapeutic and vaccine design to counter evolving threats. This Special Issue equips researchers with computational and experimental tools for pathogens such as SARS-CoV-2 variants, influenza H5N1, Mpox, and HFMD enteroviruses, fostering proactive global surveillance (Table 1). Contributions highlight hybrid workflows preempting diagnostic failures—such as in the 2025 avian flu surges and HFMD pediatric strains—while promoting equitable strategies worldwide. This Special Issue promotes sustained in silico/wet-lab synergy, enhancing resilience against RNA virus mutations amid urbanization and travel.

## Figures and Tables

**Table 1:** Conserved molecular targets versus variant challenges: examples of strategies from this Special Issue.

Pathogen	Conserved Target	Variant Challenge	Strategy Highlighted
**SARS-CoV-2**	Virus–raft interactions	Spike mutations	NTD–antiviral flexible docking [5]
**SARS-CoV-2**	CXCL10/IL-32/PRF1 T-cell signature	SARS-CoV-2 vs. other respiratory viruses	Rapid blood-based diagnosis via RT-qPCR [8]
**Mpox**	Conserved genome segments	Clade IIb mutations	In silico PCR revalidation [16]
**HFMD**	EV-A71/CV-A16 primers	Epidemic surges	Genomic surveillance [17]
